# Intelligent system based on multiple networks for accurate ovarian tumor semantic segmentation

**DOI:** 10.1016/j.heliyon.2024.e37386

**Published:** 2024-09-03

**Authors:** Mohamed El-khatib, Dan Popescu, Oana Teodor, Loretta Ichim

**Affiliations:** a National University of Science and Technology POLITEHNICA Bucharest, Bucharest, Romania; b “Ștefan S. Nicolau” Institute of Virology, Bucharest, Romania

## Abstract

Ovarian tumors, especially malignant ones, represent a global concern, with increased prevalence in recent years. More accurate medical support systems are urgently needed to support medical staff in obtaining an efficient ovarian tumors diagnosis since detection in early stages could lead to immediately applying appropriate treatment, and implicitly improving the survival rate. The current paper aims to demonstrate that more accurate systems could be designed by combining different convolutional neural networks using different custom combination approaches and by selecting the appropriate networks to be involved in the ensemble model to achieve the best performance metrics. It is essential to understand if combining all experimented networks or only the best-performing ones could always lead to the most effective results or not. The current paper is structured in three main phases. The first step is to propose the individual networks involved in the experiments. Five DeepLab-V3+ networks with different encoders (ResNet-18, ResNet-50, MobileNet-V2, InceptionResNet-V2, and Xception) were used. In the second step, the paper proposes a custom algorithm to combine multiple individual semantic segmentation networks, while the last step describes the iterative selection approach for selecting all individual networks to be combined so that the most accurate ensemble is obtained. The system performing semantic segmentation for different types of ovarian tumors, covering both benign and malignant ones, achieved 91.18 % Intersection over union (IoU), thus overperforming all individual networks. The proposed method could be extended so that more powerful deep learning models could be used.

## 1 Introduction

Ovarian tumors represent abnormal growths that can originate from the neoplastic proliferation of any type of ovarian cell. Most commonly, primary ovarian tumors involve the epithelial cells covering the surface of ovaries, while germ cells or sex cord-stromal cell proliferation are rarer, but there also exist secondary ovarian metastases from cancer of another organ. Each of the main types of ovarian tumors has various subtypes, resulting in heterogeneous lesions that can be identified by pelvic ultrasound and sometimes are difficult to differentiate [1]. Mainly they are classified into benign and malignant forms. According to Ref. [2], benign tumors with regular menses occur in 30 % of females, ones with irregular menses in 50 % of females and a large amount of them usually resolve over time, since they are functional. Based on some statistics from the same study, 70 % of these masses represent ovarian cysts, 24 % functional cysts, and 6 % malignant ones. There is a very high probability that some of the ovarian tumor masses transform into malignant ones over time. Thus, early, and correct detection of those kinds of tumor masses represents a very important aspect when it comes to mortality decrease and treatment costs, and efficient intelligent systems based on artificial intelligence are needed.

The classification of ovarian tumors as benign, borderline, or malignant is certain by histological examination after surgery, but there are ultrasonographic characteristics used by experimented examiners to classify ovarian tumors preoperatively. Ultrasound classification systems can guide the ultra-sonographers evaluating an ovarian tumor and predictor models are constantly refined to improve the detection of malignancy [3]. The Simple Rules from the International Ovarian Tumor Analysis group is a widely used tool. The features predicting a benign tumor (B features) include: unilocular cyst (B1), solid component measuring <7 mm in diameter (B2), presence of acoustic shadows (B3), smooth multilocular tumor with maximum diameter being <10 cm (B4), and absence of detectable color Doppler signal (B5). On the other hand, the Simple Rules for predicting a malignant tumor (M features) are as follows: irregular solid tumor (M1), ascites (M2), papillary structures (M3), irregular multilocular mass measuring >10 cm in diameter (M4), and strong color Doppler signal (M5). When the Simple Rules are applied, tumors that exhibit only B features and those that only exhibit M features are classified as benign and malignant tumors, respectively. If one or more B rules apply, and no M rules apply, the mass is classified as benign. The mass is considered inconclusive if both B and M rules apply, or if none of the rules apply [4].

Therapeutic intervention differs for benign and malignant ovarian tumors and sometimes even among benign tumors. Most of the tumors that appear benign can be monitored by follow-up with variable frequency, avoiding unnecessary surgery. Malignant tumors are rare, but the mortality rate of ovarian cancer remains the highest of gynecologic malignancies despite complex treatment, stressing the importance of early and accurate diagnosis [5].

Since some imaging features overlap among different types of tumors, novel solutions become attractive to improve the classification of ovarian masses. For instance, multilocular cysts have more than one septum or thin strand of tissue between internal surfaces and this category includes many benign entities such as most mucinous cystadenomas, some endometriomas, thecomas, and mature cystic teratoma, but also include mucinous borderline malignancies [6].

Early detection of ovarian cancer could increase the 5-year survival rate significantly, from 3 % in stage IV to 90 % in stage I [7]. In the absence of an efficient screening method, the need for an intelligent system becomes more and more necessary. Radiologists, as main specialists, usually perform visual analysis and interpretation of medical images for ovarian tumor localization, classification, and staging. Of course, there might be lots of cases where some radiologists could have a different outcome than others when it comes to patients with specific or complex cases. Therefore, because of the manual analysis process, and different radiologists' opinions, it might result in a final misclassification of the ovarian tumor or its stage. Therefore, there is a need for an effective intelligent support system that takes into consideration the radiologists’ experience and other past cases to efficiently provide a diagnosis.

Classic machine-learning approaches were successfully used for ovarian tumor classification. For example [8], used KNN (K-Nearest Neighbor) and SVM (Support Vector Machine) to classify ovarian cancer. However, Deep learning approaches, where incoming information is processed in a similar way to the human brain, frequently demonstrated superior performance in a wide range of tasks. Deep learning approaches were applied for imaging methods of ovarian tumors, as well as histopathological images, biomarkers, or gene expression [9]. The use of efficient intelligent systems based on deep learning for ultrasound images proves to be attractive for reducing misdiagnosis of malignant ovarian tumors, as well as avoiding high-cost imaging modalities or unnecessary surgery for benign tumors. The main advantages of deep learning approaches are characterized both by the possibility of processing large amounts of data, thus producing an effective and fast diagnosis, as well as the early detection of ovarian carcinoma, increasing treatment success [9]. Deep learning algorithms enabling image analysis, recognition, and segmentation are based on CNN (convolutional neural networks). Nowadays, such CNNs can detect, classify, or even segment ovarian tumors, allowing proper diagnosis and treatment, and thus increasing survival rate. Lots of recent papers, using deep learning approaches in the ovarian tumor diagnosis field, aim to focus on two main classes: benign, and malignant tumors. The focus usually is to perform either image classification, tumor detection, or tumor segmentation. Image classification labels the image containing the ovarian tumor while tumor detection or segmentation offers a more detailed view to radiologists, which could then make a more advanced and accurate final decision. Deep learning approaches were applied for imaging methods of ovarian tumors, as well as histopathological images, biomarkers, or gene expression [9]. The use of efficient intelligent systems based on deep learning for ultrasound images proves to be attractive for reducing misdiagnosis of malignant ovarian tumors, as well as avoiding high-cost imaging modalities or unnecessary surgery for benign tumors.

Semantic segmentation networks bring more value since they rely on each pixel classification, providing a color (label) for each, thus including classification, detection, and segmentation advantages. The authors [10] analyzed the performance of different DeepLabV3+ and FCN (Fully Convolutional Networks) semantic segmentation networks for benign ovarian tumor detection where DeepLabV3+ networks were proved to perform much better than FCNs. Such papers highlight the advantages of semantic segmentation networks over other CNNs since the outcome is represented not only by a simple tumor segmentation but also by labeling each pixel and covering multiple types of ovarian tumors, even when referring to the same image. Another important advantage is represented by the possibility to exactly identify, by labeled pixels, the contour and even the dimension of each tumor, thus illustrating a more detailed and exact view, and enabling a more efficient and even personalized treatment.

Deep learning ensemble models based on decision fusion blocks obtained remarkable results in recent years. These systems have the big advantage of considering the output prediction of each deep learning model, however, the accuracy of the final output depends on the implementation of the decision block [11]. proposed three ensemble models (majority voting model, confidence score-based model, and weighted confidence score-based model). All three classification models achieved good results, with the third one being the best performing one. The ensembles contained the first three best-performing convolutional neural networks out of ten well-known ones.

The current paper's goal is to propose an efficient collective intelligent semantic segmentation system, able to segment both benign and malignant tumors efficiently, based on three main steps. The first step is to experiment with individual DeepLab-V3+ semantic segmentation networks. The second step is to propose a network combination algorithm, like a soft voting decision block, based on each network performance for each class. The last step describes an iterative approach of experimenting with all network combination types until the segmentation errors are minimized and the performance metrics converge to the optimum ones. The resulting ensemble model, respecting the above criteria, would be considered the most accurate one.

In addition to the introduction, the paper has the following sections. Chapter 2 (Related Works) investigates the most relevant research papers in the field. Chapter 3 (Materials and Methods) presents the dataset, neural networks, software, and metrics used. It then continues with presenting the proposed method design and implementation details. Chapter 4 (Experimental Results) presents the results obtained for each network and the proposed system. The paper continues with Discussions (Chapter 5) related to the proposed system, comparing it with existing papers/methods, and ends with Conclusions where advantages and possible future improvements are presented.

## 2 Related works

During the previous years, important research studies have tried to tackle the medical diagnosis of ovarian tumors with different novel solutions. An example of such a recent research paper is [12] which relies on artificial intelligence techniques. Another example is [13] where authors proposed a novel variation of CNN architecture comparing the performance results with those of several state-of-the-art ILSVRC winning architectures. Other recent studies are based not only on deep learning methods but also on a hybrid approach. For example [14], proposed an ovarian tumor detection system based on inverted fuzzy C-Means clustering for CT image segmentation and a deep quantum CNN for the tumor detection itself, a system that outperforms other state-of-the-art methods. There are also other research papers in the same domain, using 3D networks for multiclass ovarian tumor detection. For instance, the study [15] proposes a novel 3D CNN-based modified bidirectional long short-term memory with pelican optimization to obtain notable results.

Other studies try to tackle the problem of poor segmentation of ovarian tumor details, an issue that existing methods have. For instance Ref. [16], solves this problem by proposing a dual encoding-based multiscale feature fusion network for efficient ovarian tumor segmentation. It comprises a dual encoding method step to extract diverse features, a multiscale feature fusion block for generating more advanced features, and a decoding stage after feature concatenation, thus capturing the valid information accurately.

The present paper proposes a collective intelligent semantic segmentation system to efficiently segment multiple ovarian tumor classes, covering many benign types as well as the most common malignant tumor (a total number of nine classes). The first class is represented by chocolate cysts (CC), benign masses representing cysts filled with menstrual blood. The prevalence of such benign tumors is 8 % in the case of reproductive age group and 18 % in the postmenopausal age group [17]. This form of ovarian endometriosis presents as a benign cystic mass filled with blood that creates low-level homogenous echoes with a typically ground-glass appearance on ultrasound [17,18]. The second class is represented by serous cystadenoma (SC), benign epithelial tumors that rarely transform into malignant ones like serous cystadenocarcinoma of the ovary. According to Ref. [19], this type of ovarian tumor is the most prevalent histological subtype of ovarian cancer which presents a serious threat to women's health. These tumors sometimes are large, filled with clear serous fluid, smooth internal surfaces, and rarely thin internal septations. The third class is represented by teratoma (T), a type of ovarian tumor representing germ cell tumors developing on the ovaries. Like SC, the majority are benign, with a small probability of transforming into malignant over time [20]. is an example of a study providing a comprehensive review of image findings and histopathological correlation between benign and malignant ovarian teratomas. The next covered class is represented by theca cell tumors (TCT) or thecomas, which are usually benign with an excellent prognosis, with malignant ones being rare. They are tumors of ovarian supportive, stromal cells that appear solid, often hypoechoic with well-defined borders on ultrasound, the malignant thecomas are very rare and usually contain granulosa cells too [21]. Simple cysts (SCH) are the next covered classes, with the most common findings, usually discovered on physical examination or imaging [22]. The next covered class is mucinous cystadenoma (MC), a multilocular cyst with smooth outer and inner surfaces, 80 % benign, 10 % borderline, and 10 % malignant [23]. MC is a cyst filled with gelatinous fluid, often multilocular with thin septations, and smooth outer and inner surfaces. The last and important covered tumor class is represented by high-grade serous cystadenoma (HGSC), a serous ovarian tumor type with the most lethal gynecologic malignancy, the ones with TP53 mutations being in 96 % of cases and showing an aggressive clinical course [24]. HGSC is a malignant tumor arising from the surface epithelium. It is the most common subtype of ovarian cancer and presents as a cystic complex mass with solid components, irregular borders, and papillary projections within the cystic or solid areas, associated with abdominal fluid and both ovaries involvement. To differentiate the different regions in the ovarian images, other than different benign and malignant tumors, the present study covers also non-tumor classes such as the normal ovary (NO) and the ultrasound background (B).

[10] aimed to experiment with a couple of semantic segmentation neural networks from DeepLabV3+ and fully convolutional network families on four varieties of benign tumors - CC, MC, T, and SCH. NO and B classes were also considered. The experiments were done using a small subset of the MMOTU (Multi-Modality Ovarian Tumor Ultrasound) dataset [25], where the segmentation performed by specialists was replicated using Matlab Image Labeler. The paper achieved quite good results for some of the covered networks.

The current study covers nine classes, including both benign and malignant ovarian tumors, obtaining notable results in terms of individual DeepLabV3+ network metrics, and proposes both an algorithm where multiple networks are fused for better results and an iterative selection approach for experimenting with different ensemble models to obtain the most effective one.

Compared with the related papers, providing an efficient medical diagnosis for ovarian tumors, the current paper proposes a global system where multiple networks work together to obtain semantic segmentation and tumor classification as the output. While [13] proposes a novel network to provide a diagnosis based on a classification task, the current paper offers a more visual diagnosis using semantic segmentation. Of course, recent hybrid systems such as [14], using both inverted fuzzy C-Means and a deep quantum CNN for the final diagnosis (two steps), are systems that could bring added value to an efficient diagnosis. A contribution of the proposed system is that multiple CNNs perform the same task (semantic segmentation) and the final output is generated based on a decision fusion block. Unlike the solution in Ref. [16], where the ovarian tumor is segmented using a multiscale feature fusion network based on dual coding, the proposed system makes a semantic segmentation of the ovarian tumor and an identification by combining several networks, thus obtaining a better result [10]. trained multiple individual networks for the semantic segmentation of benign ovarian tumors and only compared them. The new contribution of the proposed system is based on a network combination approach and covers malignant ovarian tumors as well.

## 3 Materials and methods

### 3.1 Dataset used

**Fig. 1:**
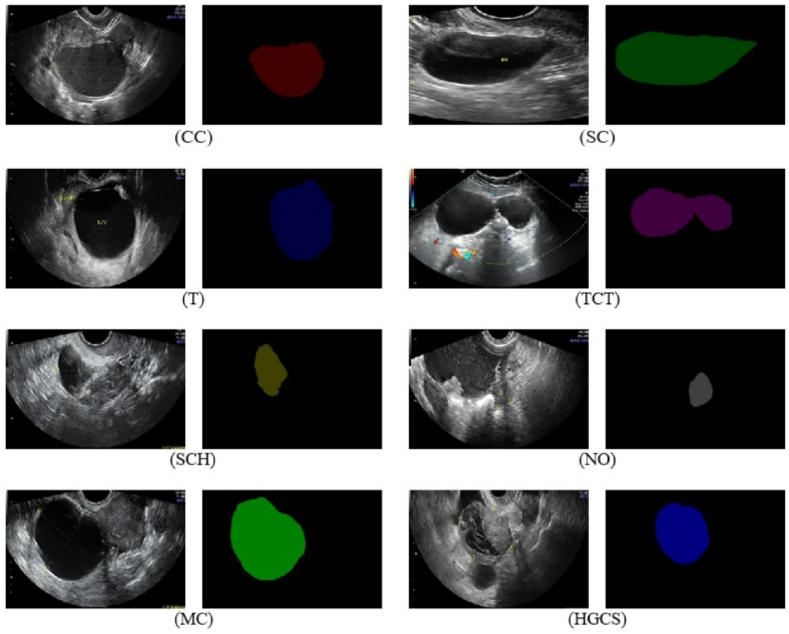
Image examples together specialist segmentations for each class from the MMOTU dataset [25] used in the current study for training individual semantic segmentation networks. Chocolate cysts (CC), serous cystadenoma (SC), teratoma (T), theca cell tumors (TCT), simple cysts (SCH), normal ovary (NO), mucinous cystadenoma (MC), and high-grade serous cystadenoma (HGSC).

The experiments in this paper are based on the MMOTU publicly available ovarian tumor ultrasound image dataset [25], composed of OTU-2d with 1469 2D ultrasound images, and OTU_CEUS (contrast-enhanced ultrasound) with 170 CEUS images. For both subsets, the dataset also provides pixel-wise semantic annotations performed by the Department of Gynecology and Obstetrics from Beijing Shijitan Hospital, Capital Medical University. The current study specifically relies on ultrasound 2D images (OTU-2d) capturing ovarian tumors of different types and normal ovaries. Fig. 1 presents two image examples (original and segmented images by specialists) for each class separately (chocolate cyst, serous cystadenoma, teratoma, theca cell tumor, simple cysts, normal ovary, mucinous cystadenoma, and high-grade serous cystadenoma). Apart from the classes presented in Fig. 1, an additional B (background)class was introduced for representing non-tumor and non-ovary-related ultrasound image parts. The dataset comes with the meaning of all eight attached colors and a metadata file labeling each image file name with the corresponding color class.

In terms of evaluating the performances of all individual networks, the dataset was split using 70%-20%–10 %. Thus, from the total number of images in the dataset, 1019 images (∼70 %) were used for the training phase, 300 images (∼20 %) were used for the testing phase, and 150 images (∼10 %) were used for the validating individual networks performance during the training phase.

The combined networks were tested on 450 images (∼30 %), obtained by merging the testing with the validation dataset from the previous approach. Class balancing was taken into consideration when splitting the dataset. The remaining problem with the current approach is that even if class balancing was considered when splitting the dataset, in the case of a semantic segmentation task/application, where the number of pixels for each class matters, there is a need to balance also the number of pixels for each class by applying image augmentation to generate more images for such classes and compensating the number of pixels. However, as it is specified in Section 6, data augmentation is subject to future work.

### 3.2 Convolutional neural networks used

Since the current study is focused on the semantic segmentation task, all networks used are based on an encoder-decoder architecture, where features are extracted by encoders, and final segmented images are provided via the decoders by up-sampling. Table 1 presents the five DeepLabV3+ networks that were considered, each with a different encoder.

**Table 1:** All semantic segmentation networks used in the current study, together with the associated alias/labels.

CNN	Alias/Label
DLV3+ (ResNet18)	CNN1
DLV3+ (ResNet50)	CNN2
DLV3+ (InceptionResNet-V2)	CNN3
DLV3+ (MobileNet-V2)	CNN4
DLV3+ (Xception)	CNN5

Even though newer and more powerful architectures were designed, the reason for using DeepLabV3+ networks is that they still provide efficient results, and some researchers are trying to improve their architecture [26]. proposed an improved version of the original network, obtaining a mean IoU between 3.07 % and 3.59 higher. ResNet-18, ResNet-50, MobileNet-V2, InceptionResNet-V2, and Xception are the backbone networks used as encoders, each of them being widely used in deep learning applications, and taking advantage of the residual blocks, thus solving the vanishing gradient problem. Of course, deeper residual networks could bring more advantages as well, however, this is subject to the future.

#### 3.2.1 DeepLabV3+

**Fig. 2:**
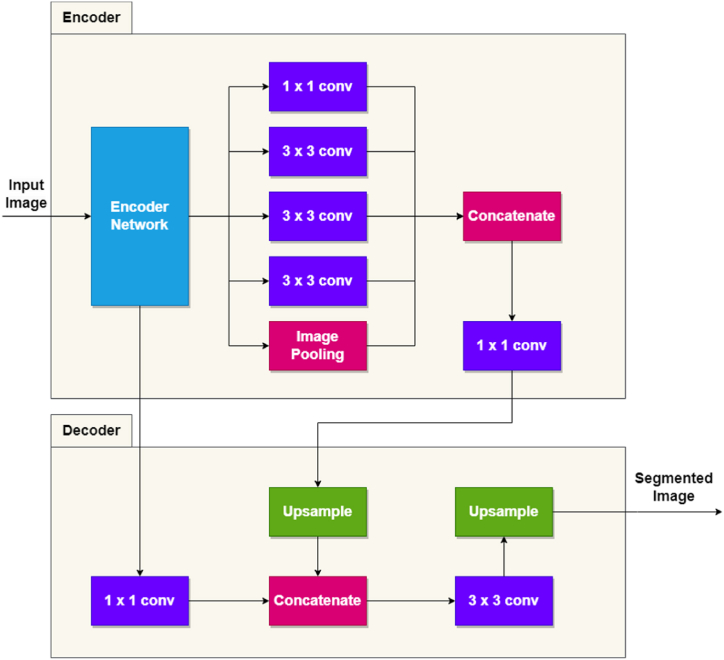
DeepLabV3+ network architecture.

DeepLabV3+ is one of the most successfully used CNNs in the semantic segmentation field. It is based on an encoder-decoder architecture, as seen in Fig. 2, adapted from Ref. [27], where the encoder encodes the information by applying atrous convolutions, at multiple scales. The final segmented image is output by other convolution operations and up-sampling by the decoder. This specific model was first introduced by Ref. [28]. All CNNs used in the current study have the same architecture as the one presented in Fig. 2, the only difference being made by the Encoder Network block, which differs from one CNN to another.

#### 3.2.2 ResNet-18

ResNet-18 is an 18-layer CNN, first introduced by Ref. [29] to overcome the vanishing gradient problem by relying on the concept of residual blocks. The network receives inputs of size 244 × 244, is pre-trained with ImageNet, contains more than 1 million images, and can classify images into 1000 categories. Residual blocks allow the networks to take the activation from one layer and feed it to another layer, no matter how deep the network, thus enabling the convolutional networks to perform better as they get deeper. Lots of the latest review research papers, such as [30], mention state-of-the-art residual networks as still being used nowadays because of the good, obtained results. For example, authors of [31], use ResNet-18 to replace the classic VGG network used as an encoder in a U-Net architecture, to efficiently segment brain tumors in MRI images.

#### 3.2.3 ResNet-50

ResNet-50 is another variant of residual networks, this time 50 layers deep. Thus, like ResNet-18, it can solve the vanishing gradient problem by using based on residual/skip connections. Pretrained on ImageNet, and being able to classify images into 1000 categories, CNN receives images of size 244 × 244 as inputs. Like ResNet-18, this network is based on a similar model presented in Ref. [29], being a model with more layers. Since it was demonstrated that residual networks perform better as they get deeper, the same applies to the current paper's experimental results as well, where DeepLabV3+ together with ResNet-50 used as an encoder, obtained better results compared to DeepLabV3+ used with ResNet-18 as an encoder. Being a state-of-the-art network, ResNet-50 is still widely used nowadays because of its efficient results in different domains. For instance Ref. [32], uses ResNet-50 for food detection. Another recent research paper is [33], where authors propose an improved ResNet-50 for efficiently identifying chicken gender.

#### 3.2.4 MobileNet-V2

Mobile networks, originally introduced by Ref. [34] are CNNs designed to be used for mobile applications and embedded vision applications. Thus, memory consumption represented the main concern. For this to happen, the concept of depth-wise separable convolutions was used to reduce both the complexity and model size. Mobile networks make use of both residual connections for solving the vanishing gradient problem and 1 × 1 pointwise convolution. MobileNet-V1 is built of 13 blocks (each containing depth-wise separable convolution and pointwise convolution). MobileNet-V2 was introduced by Ref. [35] and came as an improved version of MobileNet-V1, where authors introduced the concept of inverted residual blocks. MobileNet-V2 is built of 17 bottleneck blocks, each containing an expansion block, a depth-wise separable convolution, and a pointwise convolution. The role of the expansion block is to allow the network to learn a richer function. MobileNet-V2 input size is 244 × 244, same as for ResNet-18 and ResNet-50. The network is widely used in recent papers. An example is [36], where MobileNet-V2 is used as an encoder in a U-Net architecture for segmenting lung tumors from CT images.

#### 3.2.5 InceptionResNet-V2

InceptionResNet-V2 is a CNN taking advantage of both inception and residual blocks, thus introducing, and making use of the concept of residual inception blocks. They were first introduced in Ref. [37]. The network receives inputs of size 299 × 299, is composed of 164 layers, and is pre-trained on the ImageNet dataset being able to classify images into 1000 categories [37]. tried multiple versions of the residual version of Inception, however only InceptionResNet-V1 and InceptionResNet-V2 were detailed in the paper, having InceptionResNet-V2 being the one with the most efficient results in terms of prediction error and InceptionResNet-V1 outperforming some of the state-of-the-art networks. The overall architecture of InceptionResNet-V2, which is like InceptionResNet-V1, the difference being made by the content of each block, detailed in Ref. [37].

#### 3.2.6 Xception

Xception is a CNN, first introduced in Ref. [38], based entirely on depth-wise separable convolution layers. The network's feature extraction part is composed of 36 convolutional layers, followed by optional fully connected layers and a logistic regression layer. The authors propose the novel network, being inspired by the Inception network, replacing Inception modules with depth-wise separable convolutions. The network input size is 299 × 299 and can classify images into 1000 categories. It outperforms Inception-V3 on the ImageNet dataset. In the current paper, a version of 71 layers deep was used.

### 3.3 Software used

All experiments in the current study were performed using Matlab, a powerful programming and numeric computing platform, usually used by scientists and engineers to develop algorithms, analyze data, create models, and provide the ability to easily transpose research ideas into real world.

**Fig. 3:**
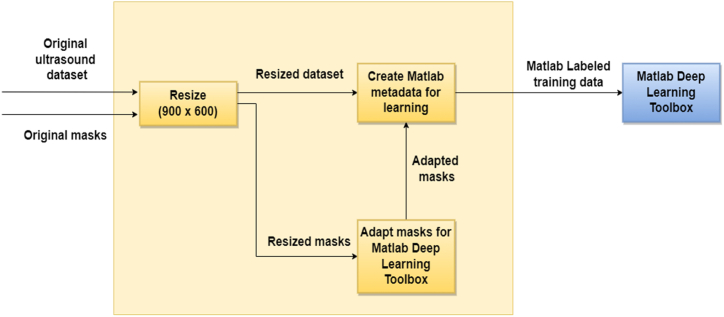
Proposed dataset preprocessing and adaptation method.

In the current paper, the original MMOTU images and the associated labeled segmentations had to be processed to work with the Matlab Deep Learning Tool. Matlab provides an image labeling tool as well, enabling users to segment and color pixels by hand, however, to take full advantage of the segmentations performed by specialists, and thus obtain better results, we created an image preprocessing and adaptation block that creates labeled data for training semantic segmentation networks using Matlab. As presented in Fig. 3, the following steps were performed:

• The original images and associated segmentations/masks were resized to (900 × 600). The reason is to keep consistency when it comes to training data size.
• Final masks/training data were created based on the original binary masks and a metadata file attached to the dataset. Original masks contain pixels with values of 0 and 1, where 0 represents the „background” and 1 represents a particular class. Class differentiation could be done only based on the metadata file, containing the mapping between a particular class and the image number.
• Other additional Matlab metadata files were created to obtain labeled data supported by Matlab for training the semantic segmentation networks.

### 3.4 Metrics used

All individual semantic segmentation networks were evaluated by F1 score (*F*1), *IoU,* precision (PREC), and sensitivity (RECALL). *F1* combines the precision and recall scores of a model using their harmonic mean*. IoU*, also known as the Jaccard similarity coefficient is used as a statistical accuracy measurement, penalizing false positives, and is a commonly used metric. PREC is used to identify positive instances out of all that were predicted as positive and is an important metric in situations where the cost of false positives is high. RECALL is used to identify positive instances among all actual positive instances in the dataset and is an important metric in situations where the cost of false negatives is high. The formulas for each are presented in (1), (2), (3), respectively (4).

(1) $$F1=\frac{2T_{P}}{2T_{P}+{F_{P}+F}_{N}}$$

(2) $$IoU=\frac{A\cap B}{A\cup B-\;\left(A\cap B\right)}$$

(3) $$PREC=\frac{T_{P}}{T_{P}+F_{P}}$$

(4) $$RECALL=\frac{T_{P}}{T_{P}+F_{N}}$$

### 3.5 Design and implementation of the proposed system

The goal of the current study is to design a collective intelligent system based on an iterative fusion of multiple semantic segmentation networks to obtain better results. To achieve this goal, both the way of computing the total number of available network combinations and the network combination approach were presented.

#### 3.5.1 Computing the total number of available network combinations

After obtaining the individual performance metrics for all the five involved semantic segmentation networks, a collective intelligent system is proposed based on iterative experiments of different network combinations and selecting the one with the minimum prediction error.

The number of possible involved network combinations *β* is given by (5):

(5) $$\beta =C_{n}^{2}+C_{n}^{3}+\ldots +C_{n}^{n}=2^{n}-\;C_{n}^{0}-\;C_{n}^{1}$$

where $C_{n}^{k}$ represents the mathematical combinations of a total number of *n* elements taken by *k*. Note that $C_{n}^{0}$ and $C_{n}^{1}$ were excluded since the experiments should include at least two and at most five combined networks.

Since *n* = 5 in the current experiment, from a total number of *β* = 27 experiments that must be performed, the one that obtains the minimum prediction error must be selected.

#### 3.5.2 Combining multiple networks approach

The algorithm requires a set of networks to combine and a set of well-defined classes. All networks are first trained individually, and their performance metrics for each class are obtained. The performance metrics for each network are represented in a dedicated matrix. The rows of each matrix represent each CNN, while the columns represent each class. The next step is to choose a performance metric matrix to be further used in the algorithm. In the current case, IoU was used as the main performance metric since it is a crucial metric for evaluating the performance of segmentation models and measuring the accuracy of an object detector on a particular dataset. Thus, the weight matrix is defined as the IoU of each network for each class. Once an input image is fed into the combined system, the same is fed into each network, each providing a decision matrix as an output. Each decision matrix contains the class for each pixel from the input image. All decision matrices are then considered when calculating the final predicted class for the pixels. The final class attached to a pixel is the one with the maximum IoU sum score obtained from each network.

The proposed algorithm for combining multiple networks is the following:

1. Consider *5* networks to combine: CNNi,iϵ{1,…,5}.
2. Consider *9* classes to investigate: Cj,jϵ{1,…,9}.
3. A performance matrix of size 5 × 9 is obtained for each metric, having the elements associated with *CNNi* and *Cj.*
4. The *IoU* performance matrix is chosen as the weight matrix [*W*], a matrix of size 5 × 9, whose element *w*(i,j) is the *IoU* obtained by *CNNi*, for class *Cj.*
5. Consider [*I*] as the input image of size (900 × 600).
6. Consider [*Di*] as the decision matrix obtained by *CNNi* for input [*I*]. Each [*Di*] has the same size (900 × 600) as the input image and represents a categorical matrix having as elements the prediction class name for each pixel of [*I*]. *F*or example, Di(m,n)=Cj represents the prediction of *CNNi* that *I(m,n)* belongs to Cj.
7. For the final pixel prediction, proceed as follows.
  7.1. A matrix of scores [*S*], of size 5 × 9 is attached to each pixel from position *(m,n)*. The elements of [*S*] are:

(6) $$S\left(i,j\right)=\left\{ \begin{array}{c} w\left(i,j\right)\;if\;Di\left(m,n\right)=Cj\\ 0,in\;rest \end{array}\right.$$

7.2. Consider that $\mathrm{Max}\left\{\sum_{i=1}^{5}S\left(i,j\right)\right\}$ correspond to *j*_*0*_. Then the predicted (segmented) image [*P*] has the element *P(m,n)* = *Cj*_*0*_ (class). The prediction is that *I(m,n)* belongs to *Cj*_*0*_.

**Fig. 4:**
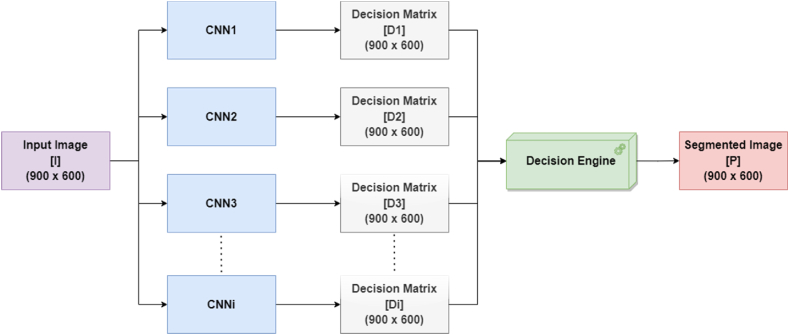
The overall architecture of the proposed network combination approach.

Fig. 4 presents an overview of the above-proposed algorithm for combining networks. The input image is fed into all individual networks, each providing a decision matrix, whose values represent the classes of each pixel. All decision matrices are fed into a Decision Engine block, responsible for executing step 7 from the algorithm presented above. The output represents the final decision matrix, having all pixels finally classified.

**Fig. 5:**
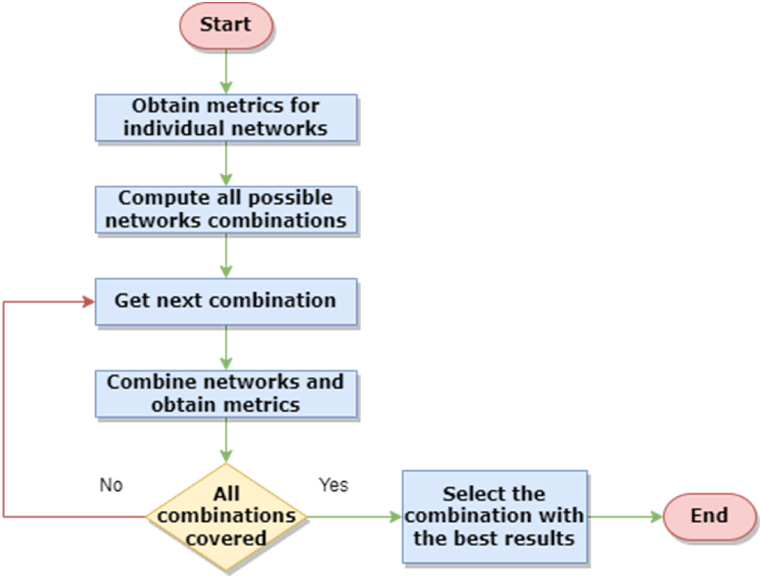
Iterative and selective networks combination approach for efficient semantic segmentation.

Fig. 5 presents the state machine of the proposed system. The first step consists of obtaining all necessary metrics associated with all individual networks. After experiments are performed and individual metrics are obtained, the next step is to compute the total number of network combinations to experiment using (5), followed by performing all network combinations using the algorithm described above. The last step consists of selecting the network combination for which the best metrics were obtained, thus concluding the iterative and selective approach.

The system is designed to obtain *IoU* for each network combination, choosing the one with the best results. Thus, the advantage of iteratively analyzing the improvement brought by some of the networks and discarding the ones negatively impacting specific combinations can be seen. The final pixel prediction is calculated using step 7 of the algorithm. The proposed method was applied for five networks; however, this could be generically applied, both in the case of a larger number and a smaller one. The presented pixel classification system is then used in a loop to determine the best pair of networks.

## 4 Experimental results

To implement the proposed system, the first step was to train all individual DeepLab-V3+ networks and obtain the IoU, F1, PREC, and RECALL performance metrics. Better performance metrics for each network lead to a positive impact on the overall combined system. The second step was to perform experiments on different network combinations, as presented in Fig. 5, and select the network combination providing the best results. The training configuration parameters are presented in Table 2.

**Table 2:** Used network training configuration parameters.

Parameter Name	Parameter Value
Optimizer	Stochastic gradients descend with momentum
InitialLearningRate	0.01
LearnRateDropFactor	0.2
LearnRateDropPeriod	3
MiniBatchSize	8
ValidationFrequency	50
ValidationPatience	5

The above configuration parameters resulted after performing some initial trial-and-error experiments and following the results obtained for each network. For example, initial experiments were performed on CNN1 and an initial number of epochs of 30 was chosen. It turned out that this number was very high and thus the model might have ended up in overfitting. To avoid overfitting in general, a small validation dataset was used to validate the networks performance on each 50'th iteration. The training process was automatically stopped in case of obtaining the same performance within the last window of 5 iterations. Different experiments with different parameters were performed for each network, to obtain the optimal training parameters for each, however, according to the experiments, it turned out that the above configuration parameters were the ones providing the best results for all of them.

Table 3 presents the percentage of the correctly identified pixels by each CNN, for each class. CNN1 obtained good results for 6 out of 9 covered classes. CNN1 doesn't perform well for HGSC, MC, and SCH, with 26.28 %. 48.41 %, and 33.13 %. One of the reasons why the 3 classes obtained lower results is because of the unbalanced number of images available in the dataset, compared with the other classes. HGSC is indeed the most dangerous ovarian tumor type, however, this could be fixed by retraining the network with more images. Data augmentation could also be applied by augmenting both the original images and the masks themselves.

**Table 3:** Percentage of correctly identified pixels.

CNN	CC	SC	T	TCT	SCH	NO	MC	HGSC	B
CNN1	94.23 %	94.49 %	91.38 %	67.71 %	33.13 %	83.24 %	48.41 %	26.28 %	98.97 %
CNN2	95.60 %	91.56 %	91.82 %	76.80 %	68.27 %	91.25 %	74.34 %	1.91 %	99.14 %
CNN3	92.35 %	92.18 %	87.69 %	21.31 %	13.95 %	84.45 %	53.38 %	1.26 %	99.07 %
CNN4	85.64 %	81.37 %	76.18 %	4.72 %	0.1 %	70.96 %	35.84 %	0.35 %	98.92 %
CNN5	86.52 %	79.21 %	79.07 %	6.52 %	7.90 %	75.54 %	61.95 %	6.65 %	99.09 %

CNN2 performed better for almost all classes, apart from HGSC, where it performed worse. Compared to CNN1, a difference between MC and SCH classes could be noticed. For CNN2, the results were 74.34 %, and 68.27 %, while for CNN2 results were 48.41 % and 33.13 %. This means that CNN1 can bring more value in terms of the HGSC class, while CNN2 can bring more value in terms of MC and SCH. This is a basic example of when some networks can compensate other networks in some areas.

CNN3 doesn't perform well on HGSC, MC, SCH, or TCT. Compared to CNN1, CNN3 performs worse for HGSC, SCH, and TCT, and better for MC. Compared to CNN2, CNN3 performs worse for all classes. Still, this network can bring some advantages for CNN1 when it comes to MC. Because of its encoder, CNN3 has more layers than the previous networks, and thus it is slower in terms of both learning and classification times. Having more layers would bring value to the proposed system itself since it could compensate by learning deeper.

Like CNN3, CNN4 does not perform well for HGSC, MC, TCT, and SCH. Of course, in terms of all these classes, CNN1 and CNN2 could compensate, as was seen in the case of CNN3 as well. However, Table 3 clearly shows the fact that the results for each class in the case of CNN4 are always lower than the ones in the case of CNN3. This might indicate that CNN4 might not bring any value when using it in combination with CNN1, and/or CNN2, and/or CNN3. On the other hand, training time was indeed much smaller for CNN4 as compared with CNN1, CNN2, and CNN3, since CNN4 is a mobile network, always used in mobile and embedded vision applications, being designed to obtain faster training and classification times by giving up some performance.

CNN5 did not perform well for HGSC, SCH, and TCT. However, CNN5 performs better than CNN4 when it comes to MC. Another advantage of CNN5 is that it performs better than CNN2, CNN3, and CNN4 when it comes to HGSC. Of course, CNN5 obtained better overall results than CNN4, and this is because CNN5 has more layers, being able to better learn deeper features.

**Fig. 6:**
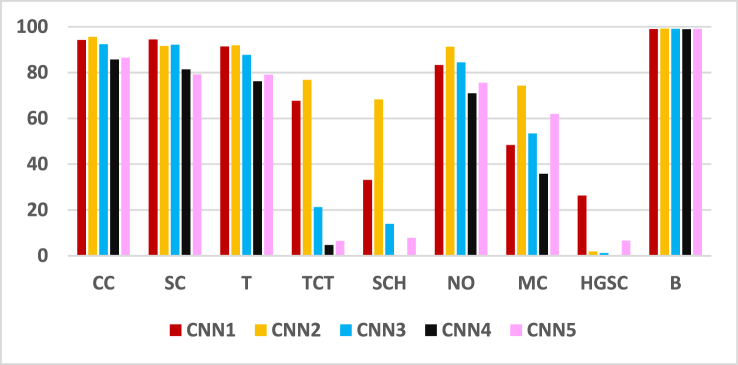
Graphical representation of the percentage of correctly identified pixels.

A graphic representation of the correctly identified pixels (in percentages) for neural networks CNN1, CNN2, CNN3, CNN4, and CNN5, corresponding to all classes (CC, SC, T, TCT, SCH, NO, MC, HGSC, and B), is given in Fig. 6.

IoU*,* F1, PREC, and RECALL metrics obtained for each network per class are illustrated in Table 4, Table 5, Table 6, Table 7. Analyzing all metrics, it turned out that the best-performing network turned out to be CNN2, while the worst-performing one turned out to be CNN4.

**Table 4:** Networks *IoU* per pixel class.

CNN	CC	SC	T	TCT	SCH	NO	MC	HGSC	B
CNN1	86.12 %	66.22 %	80.79 %	53.28 %	28.71 %	70.19 %	46.04 %	24.82 %	97.89 %
CNN2	87.79 %	79.01 %	83.69 %	54.42 %	57.75 %	75.34 %	67.84 %	1.91 %	98.23 %
CNN3	83.68 %	65.63 %	69.97 %	18.82 %	12.78 %	59.88 %	46.38 %	1.26 %	97.76 %
CNN4	71.16 %	54.93 %	56.71 %	4.41 %	0.1 %	52.24 %	26.50 %	0.35 %	96.79 %
CNN5	69.17 %	63.03 %	61.38 %	5.98 %	7.13 %	58.26 %	42.85 %	6.21 %	97.27 %

**Table 5:** Networks *F*1 score per pixel class.

CNN	CC	SC	T	TCT	SCH	NO	MC	HGSC	B
CNN1	92.54 %	79.68 %	89.38 %	69.52 %	44.61 %	82.48 %	63.05 %	39.77 %	98.93 %
CNN2	93.53 %	88.27 %	91.12 %	70.49 %	73.22 %	85.94 %	80.84 %	3.74 %	99.11 %
CNN3	91.11 %	79.17 %	82.33 %	31.67 %	22.66 %	74.91 %	63.37 %	2.49 %	98.87 %
CNN4	83.15 %	70.91 %	72.38 %	8.45 %	0.20 %	68.63 %	41.89 %	0.70 %	98.37 %
CNN5	81.77 %	77.32 %	76.07 %	11.28 %	13.31 %	73.63 %	59.99 %	11.69 %	98.62 %

**Table 6:** Networks precision per pixel class.

CNN	CC	SC	T	TCT	SCH	NO	MC	HGSC	B
CNN1	94.22 %	94.49 %	91.99 %	73.47 %	63.76 %	81.71 %	82.91 %	5.06 %	98.90 %
CNN2	95.60 %	91.71 %	92.80 %	78.48 %	75.21 %	87.19 %	86.16 %	8.99 %	99.07 %
CNN3	92.34 %	92.18 %	88.84 %	39.29 %	35.03 %	77.61 %	72.23 %	5.30 %	98.65 %
CNN4	85.63 %	81.84 %	80.14 %	12.18 %	0.35 %	68.82 %	45.58 %	0.92 %	97.82 %
CNN5	86.52 %	79.77 %	79.48 %	18.21 %	13.08 %	72.84 %	55.35 %	14.66 %	98.14 %

**Table 7:** Networks recall per pixel class.

CNN	CC	SC	T	TCT	SCH	NO	MC	HGSC	B
CNN1	90.91 %	68.89 %	86.89 %	65.98 %	34.30 %	83.26 %	50.86 %	3.27 %	98.97 %
CNN2	91.55 %	85.1 %	89.5 %	63.97 %	71.31 %	84.71 %	76.13 %	2.35 %	99.13 %
CNN3	89.91 %	69.38 %	76.70 %	26.52 %	16.74 %	72.38 %	56.44 %	1.62 %	99.07 %
CNN4	80.81 %	62.55 %	65.98 %	6.40 %	0.14 %	74.2 %	38.75 %	0.50 %	98.92 %
CNN5	77.51 %	75.01 %	72.94 %	8.17 %	13.61 %	74.42 %	65.47 %	9.72 %	99.08 %

**Fig. 7:**
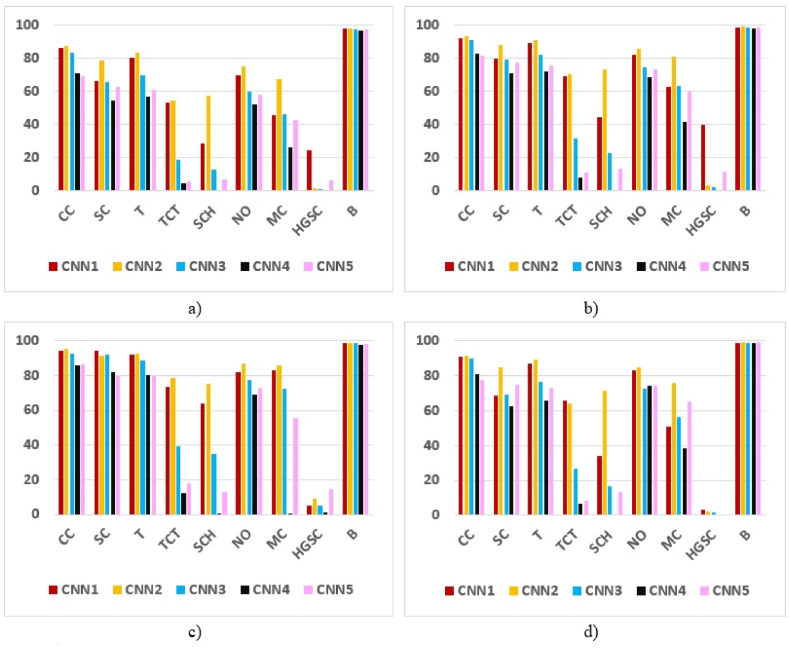
Synthetic graphical representation of network metrics per pixel class *IoU*. (a), *F*1 score (b), Precision (c), and Recall (d).

A graphic representation of the performance metrics for neural networks CNN1, CNN2, CNN3, CNN4, and CNN5 (in percentages), corresponding to all classes (CC, SC, T, TCT, SCH, NO, MC, HGSC, and B), is given in Fig. 7. Thus, IoU is represented in Fig. 7a., F1 Score - in Fig. 7b, Precision - in Fig. 7c, and Recall – in Fig. 7d. Like in Fig. 6 it can be observed that the worst results are obtained for the classes HGCS, SCH, and TCT. Class B, with the best results, is an exception (many black pixels).

Table 8 illustrates the benefits of the outcome of the combined DeepLabV3+ networks using the proposed method. Five network combinations achieve better results than the most performing individual network, in terms of *IoU* metric. An important note is that weighted *IoU* was used to compensate for the fact that the current dataset used for training is not balanced in terms of the number of pixels per class. Balancing the classes used by the current dataset could improve both the performances of the individual networks and the intelligent system; however, this was not in the scope of the current paper and could of course be subject to future implementation. The main goal was to prove that the current proposed method of combining/fusing multiple networks and selecting the better one would bring much more value than each network. Table 8 illustrates that the most-performing network combination is the one composed of the first 3 most performing individual networks: CNN1, CNN2, and CNN3, with an overall *IoU* of 91.18 %. The next performing network combination involves the first two most performing individual networks with an overall *IoU* of 88.94 %. It can also be observed that even CNN3, having overall metrics lower than CNN1 and CNN2, could bring significant improvements for the overall system metrics, thus increasing the *IoU* by ∼2.24 %. Table 8 also illustrates that the system obtains worse results when CNN4 comes into place, however, there are still two cases where combining some of the networks with CNN4 provides better results than the most performing individual network.

**Table 8:** Top 5 best-performing network ensembles versus the best-performing individual network.

Network	*IoU*
CNN1 + CNN2 + CNN3	91.18 %
CNN1 + CNN2	88.94 %
CNN1 + CNN2 + CNN3 + CNN5	88.61 %
CNN1 + CNN2 + CNN3 + CNN4 + CNN5	88.60 %
CNN1 + CNN2 + CNN3 + CNN4	86.71 %
CNN2	84.58 %

**Fig. 8:**
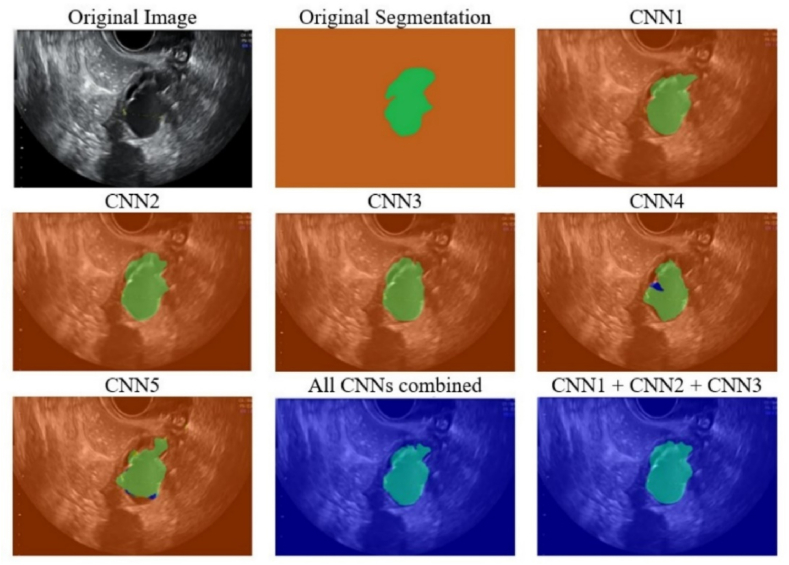
Experimental results in the case of a Chocolate Cyst for individual networks and the global system.

Fig. 8 presents the original image, original segmentation, all individual network segmentations, and two of the best-performing network combinations segmentations for CC. The orange color represents the background, while the green color represents the CC lesion itself. Even from the visual point of view, it can be observed that each network could add some value when it comes to small details, such as edge contours, etc. It can also be seen that CNN4 and CNN5 perform wrong predictions in the case of some pixels (blue ones), thus confirming why combining networks with CNN4 and CNN5 could result in a less efficient system.

By comparing the original segmentation with the one that resulted from CNN2, a small deviation on the upper part of the lesion can be observed, where a couple of B pixels are confused with CC pixels. In the case of the CNN1 + CNN2 + CNN3 + CNN4 + CNN5 combination, CNN1 and CNN4 mitigate the segmentation error, bringing it to a more correct version. The same could be observed for the lower left side edge as well. In the case of CNN1 + CNN2 + CNN3, the best-performing network, results are much closer to reality and thus the upper side, the lower left side, and the upper right side are predicted better.

## 5 Discussions and limitations

The current paper presented a novel solution for obtaining efficient ovarian tumor semantic segmentation results. The proposed system consists of four important steps: obtaining metrics for all involved networks, calculating all network combinations, performing experiments for each combination, and selecting the most performing network combination according to its associated *IoU.* At least five network combinations performing better than the most performing individual network (CNN2) were presented. The network combination providing the best results was the one combining CNN1, CNN2, and CNN3 with an *IoU* difference of 6.1 %, compared to CNN2. Thus, it was demonstrated that CNN3, with poorer performance than CNN1, and CNN2, compensate for the overall accuracy of the final system.

Table 9 presents an overall comparison between the current research and similar ones. Each of them presented good results in the current field. *IoU* is a crucial metric for semantic segmentation tasks and is used for comparison with other papers, since it provides the exact deviation of the predicted mask, compared to the originally segmented one. One of the selected papers used CT images, while most used ultrasound images. [10,25], and [39] used the same dataset, originally introduced by Ref. [25], with an important note that [39] improved the segmentation results by removing noise and additional medical-related object markers from the original dataset.

[25]implemented multiple networks for ovarian cancer semantic segmentation on the MMOTU dataset, out of which DANet and SegFormer present the most remarkable results in terms of *IoU.*
[40]performed automatic ovarian cancer detection and segmentation using a custom multitask model based on a custom network named YOLO-OCv2 which is based on YOLOv5. The paper achieved an important *IoU* of 89.88 % for the segmentation task.
[10]compared a couple of semantic segmentation neural networks from DeepLabV3+ and fully convolutional network (FCN) families on benign ovarian tumors. All experiments were done on a small subset of the MMOTU dataset [25], with the segmentation being done by specialists and the important mention that segmentation data is being manually replicated in Matlab. The paper achieved good results for some of the covered networks in terms of *IoU* metric. In terms of DeepLabV3+ networks, the conclusion was that DeepLabV3+ showed the most efficient results, as compared with FCN. The current paper brings more realistic value when it comes to covering a larger variety of ovarian tumor classes including malignant ones. More than that, the current study focused on obtaining better individual network metrics as well, thus positively impacting the final intelligent system.

**Table 9:** Comparison with other similar research papers.

Network/reference	IoU	Image Type
**CNN1 + CNN2 + CNN3 (ours)**	**91.18 %**	Ultrasound
DANet [25]	89.97 %	Ultrasound
SegFormer [25]	89.88 %	Ultrasound
Multitask model based on custom YOLO-OCv2 custom network [40]	89.63 %	CT
DeepLabV3+ and ResNet-50 as encoder [10]	83.99 %	Ultrasound
U-Net [39]	76.06 %	Ultrasound

The main difference between the architectures used in the above comparison table and the currently proposed one is that the current one uses an ensemble approach. The main advantage of the ensemble models is that multiple networks are considered to obtain a more accurate output. There are also other papers using ensemble models in ovarian cancer fields, however, they are mainly oriented to image classification rather than segmentation tasks. Examples are [11], and [41]. [11] proposed three ensemble models for accurately distinguishing benign from malignant ovarian tumors. The authors experimented with ten well-known CNNs and selected the best three performant ones to build the ensemble models. The first ensemble model was based on majority voting, the second represented a confidence score-based model, and the third represented a weighted confidence score-based model. The best-performing ensemble model turned out to be the weighted confidence score-based model. The final ensemble model obtained a final accuracy of 92.19 ± 2.88 % [41]. proposed a similar ensemble model, based on a soft voting scheme of averaging the probabilities of the involved models (VGG16, ResNet-50, and MobileNet) to classify between benign, inconclusive, and malignant tumors, and achieved 97.1 % SEN, and 93.7 % SPEC.

The current study proposed an ensemble model, combining multiple semantic segmentation networks to obtain a more accurate semantic segmentation of different ovarian tumors. A main advantage of the proposed system, compared to other existing models, is that the decision fusion block acts on each pixel output for each network and outputs a final predicted pixel based on the segmentation performance of each network, for each class.

A limitation of the existing ensemble models is that they either involve all experimented networks or the first best-performing ones. The advantage of the current proposed system is that, besides the custom networks combination approach, it makes use of an iterative networks combination selection approach, where, starting from a random network combination, experiments are performed on the next ones, until the performances converge to the optimal ones.

One limitation of the current proposed system is related to unbalanced classes. The current study used the MMOTU dataset, a relatively unbalanced dataset considering the number of images for each class. Another limitation of the current system consists of the number of experiments needed to obtain the optimal network combination configuration.

## 6 Conclusions and future work

The current paper presented a novel method, iteratively combining multiple CNNs, and selecting the most optimal configuration, for segmenting different types of ovarian tumors from ultrasound images. The proposed method achieved better results than the ones obtained for each network, showing that fusion of multiple networks decisions could bring more value in terms of final image segmentation. The proposed approach obtained very good results even if the current experiments were performed using relatively small networks. By using the same approach, the performance could be improved by using other recent networks such as different U-Net variants.

An important future improvement would be to implement data augmentation methods to obtain a balanced dataset, especially when it comes to HGSC and SCH classes, and thus increasing individual network metrics, which would automatically lead to an even more efficient ensemble model. Another important future improvement would be to implement both noise and redundant medical-related markers removal to improve the overall system metrics. Since another limitation of the current system is related to the large number of experiments needed to obtain the optimal network combination configuration, a future improvement would be to find a solution to reduce this large amount of time by proposing a network preselection method for the early removal of redundant networks. Another future and important improvement would be to adapt the same algorithm using other performance metrics when combining individual networks.

## Data availability statement

Not applicable.

**Table:** Abbreviations:

Abbreviation	Description
ACC	Accuracy
B	Background
CC	Chocolate Cyst
CNN	Convolutional Neural Network
FCN	Fully Convolutional Network
HGSC	High-Grade Serous Cystadenoma
IoU	Intersection Over Union
MC	Mucinous Cystadenoma
MMOTU	Multi-Modality Ovarian Tumor Ultrasound
NO	Normal Ovary
O	Ovary
PREC	Precision
RECALL	Sensitivity
SC	Serous Cystadenoma
SCH	Simple Cyst
SEN	Sensitivity
SPEC	Specificity
T	Teratoma
TCT	Theca Cell Tumor

## CRediT authorship contribution statement

**Mohamed El-khatib:** Writing – original draft, Software, Resources, Methodology, Conceptualization. **Dan Popescu:** Writing – review & editing, Validation, Supervision, Methodology, Conceptualization. **Oana Teodor:** Writing – original draft, Investigation. **Loretta Ichim:** Writing – review & editing, Validation, Methodology, Formal analysis.

## Declaration of competing interest

The authors declare that they have no known competing financial interests or personal relationships that could have appeared to influence the work reported in this paper.
